# Efficient Coding in Visual Working Memory Accounts for Stimulus-Specific Variations in Recall

**DOI:** 10.1523/JNEUROSCI.1018-18.2018

**Published:** 2018-08-08

**Authors:** Robert Taylor, Paul M. Bays

**Affiliations:** Department of Psychology, University of Cambridge, Cambridge CB2 3EB, United Kingdom

**Keywords:** efficient coding, population coding, visual working memory

## Abstract

Recall of visual features from working memory varies in both bias and precision depending on stimulus parameters. Whereas a number of models can approximate the average distribution of recall error across target stimuli, attempts to model how error varies with the choice of target have been *ad hoc*. Here we adapt a neural model of working memory to provide a principled account of these stimulus-specific effects, by allowing each neuron's tuning function to vary according to the principle of efficient coding, which states that neural responses should be optimized with respect to the frequency of stimuli in nature. For orientation, this means incorporating a prior that favors cardinal over oblique orientations. While continuing to capture the changes in error distribution with set size, the resulting model accurately described stimulus-specific variations as well, better than a slot-based competitor. Efficient coding produces a repulsive bias away from cardinal orientations, a bias that ought to be sensitive to changes in the environmental statistics. We subsequently tested whether shifts in the stimulus distribution influenced response bias to uniformly sampled target orientations in human subjects (of either sex). Across adaptation blocks, we manipulated the distribution of nontarget items by sampling from a bimodal congruent (incongruent) distribution with peaks centered on cardinal (oblique) orientations. Preadaptation responses were repulsed away from the cardinal axes. However, exposure to the incongruent distribution produced systematic decreases in repulsion that persisted after adaptation. This result confirms the role of prior expectation in generating stimulus-specific effects and validates the neural framework.

**SIGNIFICANCE STATEMENT** Theories of neural coding have been used successfully to explain how errors in recall from working memory depend on the number of items stored. However, recall of visual features also shows stimulus-specific variation in bias and precision. Here we unify two previously unconnected theories, the neural resource model of working memory and the efficient coding framework, to provide a principled account of these stimulus-specific effects. Given the importance of working memory limitations to multiple aspects of human and animal behavior, and the recent high-profile advances in theories of efficient coding, our modeling framework provides a richer, yet parsimonious, description of how orientation encoding influences visual working memory performance.

## Introduction

Recent investigations into the nature of working memory representations have focused on the empirical distributions of error observed in analog recall tasks. Different theoretical models have been put forward that reproduce these patterns of error with varying degrees of success (Zhang and Luck, 2008; Bays et al., 2009; Fougnie et al., 2012; van den Berg et al., 2012, 2014; Bays, 2014; Oberauer and Lin, 2017). However, all these models have shared the assumption that there is no stimulus-specific variation in the fidelity of stored feature values, e.g., that the distribution of recalled orientations around the true target orientation is the same regardless of whether the target is vertical, horizontal, or oblique.

In contrast, empirical studies have reported stimulus-specific variations in the recall of colors (Bae et al., 2014; Hardman et al., 2017) and orientations (Pratte et al., 2017). In particular, variations in orientation recall mimic psychophysical findings showing superior discrimination for cardinal orientations over obliques (Appelle, 1972; Girshick et al., 2011) and response biases away from cardinal axes (de Gardelle et al., 2010; Tomassini et al., 2010). These perceptual anisotropies are thought to arise from inhomogeneities in the organization of orientation-selective neurons, providing a putative biological basis for stimulus-specific variation. Comparatively, current efforts to accommodate stimulus-specific effects within models of visual working memory have relied on *ad hoc* amendments to existing model frameworks [the slots-plus-averaging model in Pratte et al. (2017); the multinomial processing tree (MPT) model in Hardman et al. (2017)]. Though these modifications provide improvements in model fit, they are unable to explain the mechanistic processes that underlie stimulus-specific variability.

One successful model of working memory errors is based upon principles of neural population coding, a fundamental process that underlies the representation of sensory information throughout cortex (Pouget et al., 2000, 2003). The model describes an input–output system whereby feature information is first encoded in the noisy firing of a population of feature-selective neurons and later decoded by reconstructing feature values from the evoked spiking activity. This framework provides an accurate description of empirical error distributions across set size manipulations (Bays, 2014, 2015) and an improvement over slot-based accounts that model response distributions as a mixture of memory-based and guessing processes (Luck and Vogel, 1997; Zhang and Luck, 2008). It has subsequently provided principled explanations for the presence of swap errors (Schneegans and Bays, 2017) and the effects of retrospective attention (Bays and Taylor, 2018) in analog recall tasks. However, despite providing a biologically plausible mechanism for recall errors, the assumption that features are encoded in a homogeneous population of neurons means it, too, is incapable of capturing stimulus-specific variations in recall.

Here our goal was to provide a principled account of stimulus-specific effects in analog recall of orientations. Our approach builds on recent theoretical work that has shown how the stimulus distribution ought to constrain the encoding and decoding stages of sensory processing (Ganguli and Simoncelli, 2014; Wei and Stocker, 2015), and is similarly based upon the principle of efficient coding, which states that neurons are optimized to represent stimuli as they occur in the natural environment (Barlow, 1961). It is well established that cardinal orientations occur more frequently than obliques in naturalistic settings (Hansen and Essock, 2005; Girshick et al., 2011). Given a parametric description of this distribution, it can then be integrated within the population coding framework. This permits the derivation of efficient population codes that allow us to capture variations in both precision and bias in analog recall tasks.

## Materials and Methods

Here we detail three existing continuous report studies to which we fit the neural resource model with the aim of validating the efficient coding account. Efficient coding produces a predictable pattern of response bias that ought to be sensitive to changes in the environmental statistics. We subsequently describe a separate experimental study that was designed to test whether response biases indeed shift in the predicted fashion when changes are made to the stimulus distribution.

### 

#### 

##### Data sets and analyses.

The continuous report task requires observers to reproduce remembered stimulus features on an analog response scale. For the continuous report of orientation, the general procedure starts by presenting an array of oriented items to be remembered, which is then followed by a blank retention interval. During the test phase, a probe display is presented that indicates which item in memory should be reproduced. The observer then uses the specified response format to rotate the probe item so that it matches the original stimulus orientation that was presented at the indicated array location. We fit data from three continuous report experiments, consisting of one data set from our own laboratory (Bays, 2014) and two data sets that have been made available by the authors (van den Berg et al., 2012; Pratte et al., 2017).

The procedural details varied across each study and are laid out in Table 1 (for further details, the reader is referred to the methods sections of the original studies). We note that to reduce computation time, we considered only a limited number of set sizes from the van den Berg et al. (2012) data set. For all analyses, the range of possible orientations [−90°, 90°) was mapped onto the circular space [−π, π) radians. Response error was defined as the angular deviation between the orientation reported by the participant and the correct target orientation. We calculated recall bias as the circular mean of response error, and recall precision as the reciprocal of the squared circular standard deviation, 1/σ^2^, as in (Bays, 2014).

**Table 1.** Experimental studies

Study	Stimulus type	Response	Set sizes	Subjects	Trials
Bays (2014), Exp. 1	Lines	Dial	1, 2, 4, 8	8	225
van den Berg et al. (2012), Exp. 2*^a^*	Gabors	Mouse/key	1, 2, 4, 8	6	320
Pratte et al. (2017)	Gabors	Key	1, 2, 3, 6	12	640

*^a^*Full dataset includes sets 1–8.

To visualize the stimulus-specific effects, we estimated bias and precision at 50 equally spaced points (bin centers) along the orientation dimension. To measure each effect, we calculated the weighted circular mean and SD (converting to precision), with weights determined by centering a von Mises kernel on each of the 50 points. In general, the raw bias curves were noisier, so we chose broader bandwidths for smoothing the bias functions (SD of kernel, *h* = 0.61) compared with the precision functions (*h* = 0.23). The smoothing procedure was applied for each observer separately. Plotted curves reflect the group means, with shaded regions corresponding to ±1 SE. Note that all model fitting used raw response data and not the smoothed data described here.

##### Neural resource model.

The neural resource model was originally instantiated using a homogeneous population of tuning functions, such that each function shared a fixed width and was translated along the feature dimension at even intervals (Bays, 2014). The key difference here is that we allow each neuron's tuning function to vary in accordance with the principle of efficient coding. To do so, we first require a prior distribution that reflects how orientations are distributed in nature. We used a bimodal distribution that peaked at the cardinal orientations. This form provides a good approximation to the distribution of orientations derived from analysis of natural images (Girshick et al., 2011; Wei and Stocker, 2015), and was defined as follows:

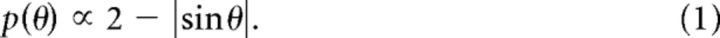
 In previous work, we assumed a homogeneous population of *M* neurons with selective orientation tuning. The average response of the *i*th neuron, with preferred orientation ϕ*_i_*, was described by a von Mises tuning function:

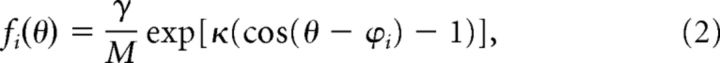
 where γ defines the total population gain and κ sets the width of each function. The preferred orientations for each neuron, ϕ*_i_*, were evenly spread throughout the orientation range to provide a dense uniform coverage. Recall that the principle of efficient coding states that sensory representations should be optimized with respect to the distribution of orientation in nature. Under the assumption that orientations are uniformly distributed, then the homogeneous population described above would be efficient. For nonuniform stimulus distributions, a configuration is required that ensures the mutual information between the external stimulus and the internal measurement is maximized. As shown by Ganguli and Simoncelli (2014) and Wei and Stocker (2015), we can approximate this optimal solution by redistributing the tuning functions using the cumulative distribution function of the prior:

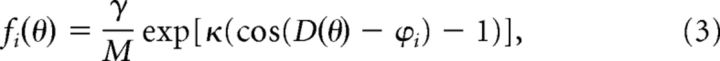
 where *D*(θ) = ∫_− π_^θ^ *p*(θ′)*d*θ′. The resultant warping of the homogeneous population satisfies the requirement that the cellular density be redistributed so that a greater proportion of the population is now devoted to encoding cardinal orientations. Additionally, the tuning functions of neurons selective for cardinals are also narrower. We refer to κ as the basis tuning width, which denotes the shared width of the homogeneous tuning functions from which the heterogeneous population is derived.

Divisive normalization (Carandini and Heeger, 2012) further scales the population activity based on the total number of memoranda, *N*. For large, homogeneous, neural populations (i.e., large *M*), the summed population activity is independent of stimulus orientation. Under these conditions, population activity may be normalized by the number of items presented on each trial to capture the effect of set size on the error distribution width. Conveniently, this fact remains true even after warping the functions: the heterogeneity in tuning functions can be viewed as a remapping of stimulus value θ in the heterogeneous population (Eq. 3) to a new stimulus value *D*(θ) in the homogeneous population (Eq. 2). Because summed population activity is constant for all θ, it must also be constant for all *D*(θ). This permits divisive normalization to proceed in exactly the same fashion as for the homogeneous population. Accordingly, the post-normalization firing rate of each neuron may be written as follows:

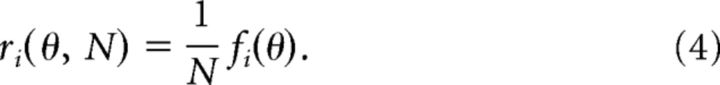
 Spiking activity was modeled by a homogeneous Poisson process such that the probability of the *i*th neuron generating *n_i_* spikes in time *T* was as follows:

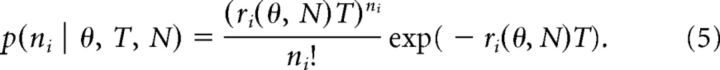
 The population response is defined by the population vector **n** = [*n*_1_, *n*_2_ …, *n_M_*]. Accordingly, the likelihood of θ with respect to **n** may be written as follows:

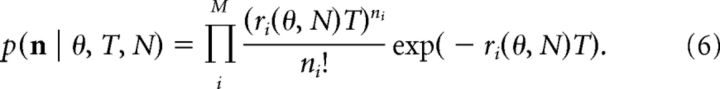
 Without loss of generality, the decoding interval *T* was fixed to unity.

In accordance with Bayesian theories of perception (Knill and Richards, 1996), we assume that the resulting efficient internal measurements are subsequently weighted by how frequently specific orientations occur in nature. The integration of these two sources of information yields a percept that reflects the visual system's best estimate of the originally presented orientation. To decode feature values from the population response **n**, we estimated the posterior mean as follows:

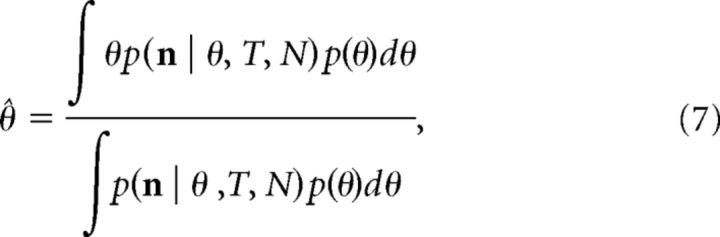
 where *p*(θ) is the stimulus prior, as defined by Equation 1. We set the population size to *M* = 100, leaving only the population gain, γ, and the tuning curve width, κ, as free parameters.

##### Slots-plus-averaging model.

The slots-plus-averaging model (Zhang and Luck 2008) proposes that item features are stored in a fixed number of memory slots, *K*. Each slot holds a single item feature with a fixed precision, but slots are treated as a quantized resource that is distributed as evenly as possible between array items, *N*. Item precision can be improved by storing and averaging across multiple copies of the same item.

Here, for consistency, we follow Pratte et al. (2017) by equating precision with the concentration of a von Mises distribution, an approximation that actually holds true only in the limit of high concentrations, in which case the von Mises distribution approximates a Gaussian and concentration becomes $\frac{1}{\sigma^{2}}$. Each item can receive one of two possible numbers of slots: $S_{low}=\left\lfloor \frac{K}{N}\right\rfloor$ or $S_{high}=\left\lfloor \frac{K}{N}\right\rfloor +1$, where ⌊*x*⌋ denotes the floor function. Given a set size of *N*, the effect of averaging is approximated by using a single von Mises distribution with a concentration κ*_n_*, which is based on the weighted average of the *S_low_* and *S_high_* concentration parameters as follows:

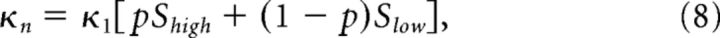
 where *p* = $\frac{K\mathrm{mod}N}{N}$ determines the probability with which an item is allocated *S_k_* slots and κ_1_ is the precision for a single slot (again, this approximation holds true only in the limit of high concentrations). When *N* ≥ *K*, then some number of items will not have access to memory. For such items, all information is lost. Should one of the non-memory items be probed, individuals are assumed to generate random responses, the proportion of which is determined by the guess rate *g_n_* = 1 − $\frac{K}{N}$.

Pratte et al. (2017) made the following modifications to allow the slots-plus-averaging model to capture stimulus-specific effects. To accommodate the effect on precision, κ_1_ was allowed to vary across orientation values using the following function:

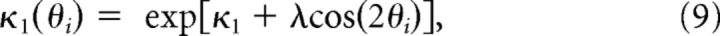
 where λ is a free parameter that controls the strength of modulation. To capture the effects on bias, the mean of the response distribution μ was allowed to vary by introducing the following function:

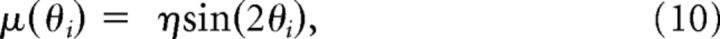
 where η is a free parameter that determines the direction and magnitude of the response bias. The complete response distribution for the slots-plus-averaging model is as follows:

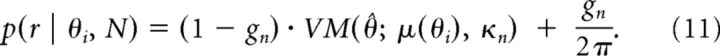
 In total, the slots-plus-averaging model requires estimation of four parameters: *K*, the number of slots; κ_1_, the precision of a single slot; λ, the magnitude of variation in precision; and η, the amplitude of bias.

##### Model fitting and comparisons.

Maximum likelihood estimation proceeded via a grid search across each model's parameter space. For the neural resource model, we searched a 100 × 100 grid with κ ranging from 0.1 to 10 (in 0.1 steps) and γ taking integer values between 1 and 100. For each parameter combination, we used Monte Carlo methods to approximate the response distribution *p*(θ̂|θ). We first defined the vector **s** = [θ_1_, θ_2_ …θ*_J_*], which represented *J* = 25 discrete and evenly spaced target orientations in the interval [−π, π). For each element in **s**, we simulated 10^4^ model responses, where the posterior mean (Eq. 7) was based on values calculated at 10^2^ evenly spaced points. We next obtained the response distribution **r** = [θ̂_1_, θ̂_2_ …θ̂*_Q_*] by calculating a histogram estimate based on *Q* = 25 equally spaced bins. Model predictions were thus defined by an **R** = *J* × *Q* stimulus-by-response matrix, with one matrix specified for each set size, *N*. Observer responses were binned in the same way. The model was compared with each observer's data separately, and the best-fitting parameters were found by maximizing the sum of the log-likelihood:

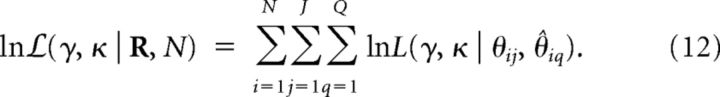
 Grid search for the slots-plus-averaging model involved searching a four-dimensional array with *K* taking integer values between 1 and 10, κ_1_ ranging from 0.1 to 10, η ranging from −1 to 1, and λ ranging from 0 to 1, each incremented in 0.1 steps. The likelihood was evaluated at the same points in the stimulus–response space, **R**, for each parameter combination, using Equation 11. The model was fit to each observer data set individually, and the best-fitting parameters were found by maximizing the sum of the log-likelihood,

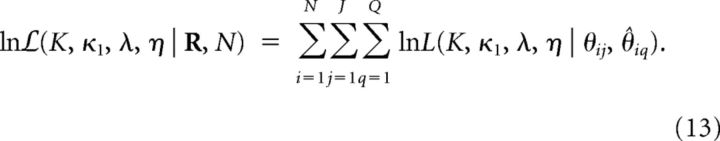
 Formal model comparison proceeded via assessment of the Akaike information criterion (AIC). The AIC quantifies fit by first assessing the model deviance and then applying a penalty term that adjusts for model complexity. Formally, AIC = −2lnℒ + 2*P*, where *P* is the number of free parameters.

For visual comparisons of the predictions made by the neural resource model with the observer response data, we simulated model predictions based on the maximum likelihood parameters. We selected 100 evenly spaced points along the orientation dimension corresponding to different possible target values and generated response distributions for each value by simulating 10^4^ model responses, i.e., decoded feature values. We then produced smaller data sets by randomly sampling values from each of the simulated response distributions. The number of samples drawn from each distribution was matched to the average number of responses per binned target value in the empirical data. This is important because estimates of precision in particular are influenced by the number of samples available. Finally, for each target value, we calculated the circular mean and SD (converting the latter to precision), thus yielding 100-point bias and precision curves. For each observer, the resampling procedure was repeated 500 times and averaged to generate subject-level predictions. The displayed model fits reflect the group-averaged functions. All model simulations, model fitting, and analyses were performed using R (R Core Team, 2016).

##### Experimental procedure.

A total of 16 participants (11 female, 5 male; age, 18–31 years) took part in the study after providing informed consent, in accordance with the Declaration of Helsinki. All subjects had normal or corrected-to-normal visual acuity. Stimuli were presented on an LCD monitor (45 × 28 cm) with a refresh rate of 60 Hz. Observers were positioned 60 cm from the screen with their head supported by a chin and forehead rest. Eye position was monitored online at 1000 Hz using an infrared eye tracker (Eyelink 1000, SR Research). Each participant took part in one of two experiments, described below.

In Experiment 1A (incongruent distribution; eight participants), trials began with the presentation of a central white fixation dot (0.25° of visual angle) and eight evenly spaced light gray dots arranged around the circumference of an imaginary circle (radius 6° against a gray background). After establishing a stable fixation within 2° of the fixation dot, a sample array consisting of four oriented white bars (1.5° × 0.1°) was presented for 500 ms (see Fig. 5*A*). Bar positions were randomly chosen from the set of eight locations. The bars were then removed for 1 s, after which a probe bar with a random orientation was presented in one of the previously occupied locations, randomly chosen. Subjects used the mouse to reorient the bar so it matched the remembered orientation of the item previously presented at the same location (the target). Responses were self-paced and registered by clicking the mouse button. Subjects were instructed to be as precise as possible. Trials where gaze deviated >2° from fixation were aborted and a new sample array presented.

Each participant completed a total of 576 trials, split into eight blocks of 72 trials each. These were defined as preadaptation (block 1), adaptation (blocks 2–7), and postadaptation (block 8). In all blocks, target orientations were sampled uniformly at random from the full range of possible orientations [−90°, 90°). During preadaptation and postadaptation blocks, nontarget orientations were also uniformly sampled; however, during adaptation blocks, nontarget orientations were sampled from a bimodal stimulus distribution with peaks centered on the oblique orientations (−45° and 45°). Specifically, the probability density was proportional to 2 −|cosθ| for orientation θ, i.e., opposite to the distribution of orientations in natural images (Girshick et al., 2011). Experiment 1B (congruent distribution; eight participants) was identical to Experiment 1A except for the nontarget sampling distribution on adaptation trials, which was a bimodal distribution with peaks centered on the cardinal orientations (0° and 90°). The probability density was proportional to 2 −|sinθ| for orientation θ, i.e., matching the distribution of orientations in natural images. After completion of all experimental sessions, participants were asked whether they had noticed any orientations occurring more, or less, frequently during the adaptation blocks. None of the participants reported noticing any changes.

##### Experimental analysis.

To minimize the contribution of noise in estimating response bias, we fit a contamination model to the data (Kennedy et al., 2017). The model assumes that circular random variables can be captured by a von Mises distribution, though some proportion of the data may reflect contaminant responses that arise from another generative process. To allow for this possibility, we included a uniform contaminant distribution and modeled the data as a mixture of these two distributions. It is important to make a clear distinction between statistical and psychological models. Mixture models of the type described above are often used to decompose data into components that are associated with some putative psychological process or operation. In the present case, however, the model is strictly a statistical approach to reducing noise in the data, and we place no psychological interpretation on the model parameters.

To capture the effects of response bias, we allowed the mean of the von Mises distribution to systematically vary along the orientation dimension. Accordingly, for each participant we defined response error from the *i*th target orientation on trial *j* as Δθ̂*_j_* = θ̂ ⦵ θ*_i_* and modeled recall errors in the following way:

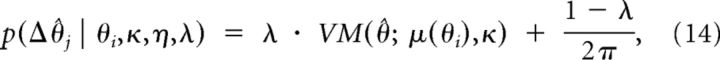
 where VM(·) is the von Mises density function, λ is the mixture weight, κ is the von Mises concentration parameter, and μ(θ*_i_*) = ηsin(2θ*_i_*). The bias parameter η controls the direction and magnitude of the bias as a function of target orientation. Positive η values indicate a repulsive bias from the cardinals, or attractive if the values are negative (η = 0 is equivalent to evaluating all errors under a zero-centered von Mises distribution). Fitting the model simply involved maximization of the summed log-likelihood lnℒ(κ, η, λ | **y**) = ∑_*j* = 1_^*N*^ln*L*(κ, η, λ | Δθ̂*_j_*), with *N* denoting the number of trials completed within each block. We used a Nelder–Mead simplex to find the best-fitting parameter values (via R's optim function), initiating searches from 100 random starting points and fitting the individual data for each block separately.

All statistical analyses were implemented using linear mixed-effects models and the lme4 package in R (R Core Team, 2016). To determine whether there was any effect on bias across the adaptation blocks, we first fit a linear mixed-effects model to the by-block parameter estimates that included only by-subject random intercepts and slopes. This null model was then compared with an augmented model that included block as a fixed effect. Model comparison proceeded via χ^2^ tests on the model log-likelihoods. Evaluation of interactive effects between sampling distribution conditions (treating experiment and block as fixed effects and including by-subject random intercepts) was based on Type III Wald *F* tests with Kenward–Roger degrees of freedom approximation.

## Results

**Figure 1.**
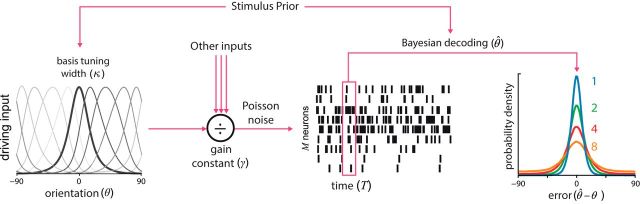
The neural resource model with efficient coding. Feature information is encoded by a heterogeneous population of orientation-selective neurons, where the shape of each tuning function was determined by the distribution of orientations in nature. The basis tuning width κ refers to the width of homogeneous tuning functions before transformation. Normalization operated across the entire population and scaled the total activity to a level determined by the gain parameter, γ. Spike generation was modeled as a Poisson process. Recall estimates are obtained by a Bayesian decoder that calculated the posterior mean based on the spiking activity over a fixed time window. Accordingly, both encoding and decoding processes rely on the stimulus distribution (the prior). Averaging over stimulus inputs, decoding produces a distribution of error (right) that is unbiased (mean zero) and increases in dispersion with set size (1–8 items shown**)**; however, the model predicts substantial variation in both bias and precision as a function of the specific stimulus encoded.

Our modeling framework assumes that feature information is represented in the spiking of orientation-selective neurons via a population code. The total activity dedicated to encoding memory items is fixed (normalized) across changes in set size. In previous work (Bays, 2014; Schneegans and Bays, 2017), the tuning functions that relate stimulus feature values to each neuron's spiking probability have been homogeneous, i.e., fixed in width and shape, and evenly distributed across the feature space. Here we allow each neuron's tuning function to vary in accordance with the principle of efficient coding, which states that sensory systems ought to devote more resources to features that occur with higher probability (Barlow, 1961). We used a bimodal stimulus distribution that peaked about the cardinal orientations to derive such a population and decoded feature information from the population activity using Bayesian inference, where the stimulus distribution acted as the prior distribution (Fig. 1; see Materials and Methods for details).

**Figure 2.**
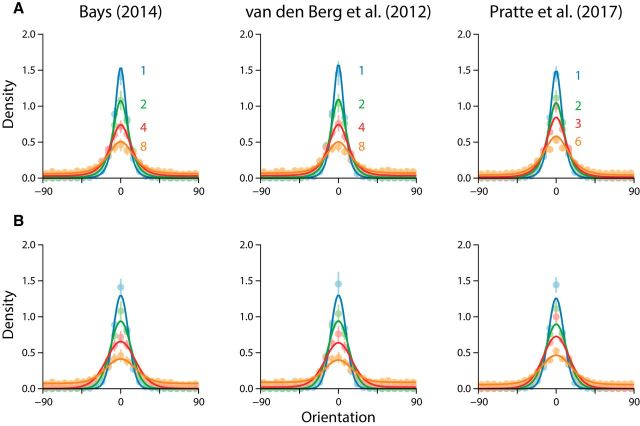
Empirical error distributions for each set size condition, collapsed across all target feature values. Averaged observer data are denoted by points (±1 SE) and model fits by corresponding solid lines. ***A***, Fits from the neural resource model. ***B***, Fits from the slots-plus-averaging model.

We fit the neural resource model to three existing continuous report data sets (van den Berg et al., 2012; Bays, 2014; Pratte et al., 2017). Each study presented arrays of oriented stimuli and required observers to reproduce the orientation of a probed stimulus (the target) on a continuous scale (see Materials and Methods for details). We first note that the mean distribution of recall errors (averaged across target orientations) displayed a typical set size effect in each study, becoming progressively broader as set size increased (Fig. 2*A*, data points). Furthermore, as noted in previous studies, the average error distributions did not follow the familiar normal distribution, but rather were leptokurtic, i.e., they had sharp peaks and long tails. These features of the error distributions were successfully captured by the neural resource model, as in previous work (Fig. 2*A*, solid lines).

**Figure 3.**
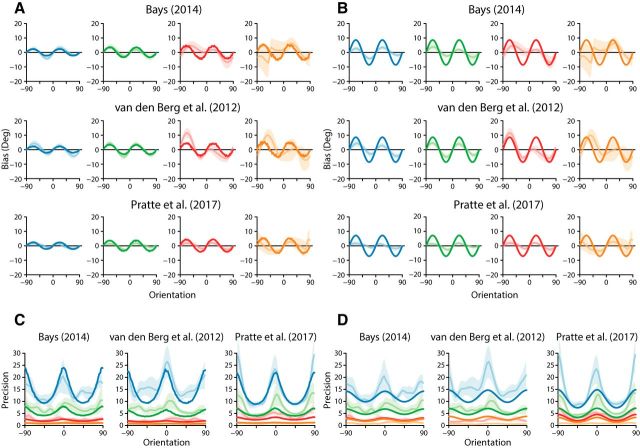
Stimulus-specific effects on bias and precision. Averaged observer data are denoted by the lighter solid lines and corresponding shaded regions (±1 SE). Averaged model fits are indicated by the darker lines. Different colors correspond to different set sizes (as in Fig. 2). ***A***, ***B***, Response biases for each set size condition within each study. ***A*** displays neural resource model fits, and ***B*** shows the slots-plus-averaging fits. ***C***, ***D***, Changes in precision for each set size condition within each study. ***C*** displays the neural resource model fits, and ***D*** shows the slots-plus-averaging fits.

We next examined how recall bias and precision varied with orientation of the target item. We found that responses were systematically biased away from the cardinal axes (0° and 90°) toward the obliques (45° and −45°; Fig. 3*A*, lighter lines). These results are qualitatively consistent with biases previously reported in psychophysical tasks (de Gardelle et al., 2010; Tomassini et al., 2010). Critically, the predictions of the neural resource model, incorporating the prior from natural images, accurately reproduced these patterns of bias (Fig. 3*A*, darker lines).

**Figure 4.**
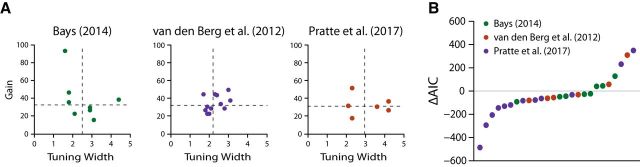
Assessment of neural resource model fits. ***A***, Individual participants' parameter estimates for the neural resource model across experiments. The dashed lines denote the median parameter values. ***B***, Individual model comparisons with the slots-plus-averaging model. Negative values indicate a preference for the neural resource model.

Within each set size, recall precision was markedly better for cardinal orientations than for obliques (Fig. 3*C*, lighter lines). This effect was most pronounced within the smaller set size conditions and attenuated for larger item arrays. The neural resource model provided a very good description of these variations in precision (Fig. 3*C*, darker lines). Finally, there was a good deal of consistency in performance across all observers, despite there being methodological differences among the studies. This is evident in the relative consistency of model parameter estimates obtained for all observers (Fig. 4*A*). Note that all the features of the data reported above were reproduced with only two model parameters: there were no parameters specifically related to the stimulus-specific variations in bias and precision, as these were a consequence of the nonuniform prior distribution.

### Comparison with a discrete capacity model

We contrasted the predictions made by the neural resource model with a recent extension of the slots-plus-averaging model. This is an example of a discrete capacity model in which visual items are stored in a small set of independent “slots,” each of fixed precision (Zhang and Luck, 2008). In this model, more than one slot can be allocated to a single item, in which case the brain holds multiple independent representations of the same visual object, which are averaged together at recall to increase precision. Yet, even with this resource-like mechanism, discrete capacity models have typically fared poorly in reproducing empirical data compared with models based on allocation of a unitary continuous resource (Bays et al., 2009; Fougnie et al., 2012; van den Berg et al., 2012, 2014; Bays, 2014; Schneegans and Bays, 2016; Bays and Taylor, 2018).

To model the effects of stimulus-specific variation, Pratte et al. (2017) merely approximated the effects using trigonometric functions chosen for their ability to describe the biases and precisions observed in their data (see Materials and Methods). Predictions of the slots-plus-averaging model for mean error distributions are shown in Figure 2*B*. While the model quantitatively reproduced the decline in precision with set size, it notably underestimated the peakedness of the error distribution, particularly at smaller set sizes. Fits to stimulus-specific bias and precision are shown in Figure 3, *B* and *D*. A consequence of efficient coding in the neural resource model is that bias and precision necessarily covary: one cannot be changed without affecting the other. In contrast, the extended slots-plus-averaging model permits bias and precision to vary independently but is constrained in that it predicts the same degree of response bias for every set size. This simplification led to poorer overall fits to observer bias (Fig. 3*B*): the model predicted a much larger degree of bias for smaller set sizes than was observed in the data, fitting only the larger set sizes with any degree of accuracy. In comparison, the neural resource model predicts an increase in response bias with set size, which seemingly allowed the model to better match the observed variation in bias magnitude across set sizes.

The extended slot-plus-averaging model also failed to accurately capture the magnitude of stimulus-specific variations in precision (Fig. 3*D*). Individual differences between model fit statistics revealed a clear preference for the neural resource model: of the 26 individual fits, 19 observers were better fit by the neural model (73%; summed ΔAIC = −943.78; Fig. 4*B*). The extended slot-plus-averaging model has four free parameters, of which two were included to capture stimulus-specific variation in bias and precision. Note that we could have compared models using the Bayesian information criterion, as in the study by Pratte et al. (2017), but we chose to use AIC as it penalizes model complexity less harshly and so is more generous to the slots-plus-averaging model.

In addition to slots-plus-averaging and variable precision models, Pratte and colleagues (2017) also considered a “hybrid model” that allowed the precision of individual slots to vary stochastically across trials. According to their description, the precision of each slot varies according to a gamma distribution, the mean of which varies as a function of set size. We found this account theoretically incoherent and antithetical to the slot concept and so did not consider this model an appropriate candidate for comparison. The original idea underlying the slot model (Luck and Vogel, 1997) was that a fixed number of items could be stored in working memory, all with a fixed high resolution. This concept was later weakened by Zhang and Luck (2008), who, in an attempt to account for the effects of set size on precision (Palmer, 1990; Wilken and Ma, 2004; Bays and Husain, 2008), allowed slots to act as a quantized resource that could be shared out between items. However, the precision of each slot remained fixed. If the fidelity of a slot were allowed to vary from moment to moment (or even as a function of set size), as proposed, then it would mean the slot concept had been abandoned in all but name.

### Experimental validation of model predictions

The results above indicate that the neural resource model, equipped with efficient coding, was able to capture many characteristics of human recall error. This in part relies on the fact that efficient coding principles can lead to biases that seemingly violate Bayesian predictions (Wei and Stocker, 2015). Bayes' theory tells us that when there is uncertainty about a stimulus based on current evidence, we should base our decision to a greater extent on prior expectations (Knill and Richards, 1996). Typically, this means biasing estimates of a stimulus feature toward values that our experience tells us occur most frequently: for orientation, this suggests that responses should be attracted toward the cardinal axes. Yet, in many instances, as here, the opposite is observed (de Gardelle et al., 2010; Tomassini et al., 2010). Nonetheless, the efficient coding account asserts that these seemingly “anti-Bayesian” biases are a consequence of incorporating the environmental distribution of stimuli into the neural code.

Specifically, these biases are a consequence of the redistribution of tuning functions toward the cardinal orientations, which results in greater uncertainty in decoding oblique than cardinal orientations. Consider a stimulus deviated a little clockwise of vertical: because tuning functions are steep and densely packed close to the vertical, the deviation of the stimulus away from vertical can be discriminated very precisely. In the other direction, toward the nearest oblique angle (45° clockwise of vertical), tuning curves are shallower and sparser, meaning there is greater uncertainty regarding the stimulus in this direction (technically, the likelihood distribution is skewed, with a longer tail in the oblique direction). The result is that a point estimate of the stimulus will be biased away from the vertical, and this effect may be strong enough to outweigh the bias toward the vertical induced by the Bayesian prior (Wei and Stocker, 2015).

If anti-Bayesian biases are indeed a result of adaptation to the stimulus environment, a very testable prediction follows: changing the environmental statistics should produce predictable shifts in response bias. Specifically, if an environment is created where, contrary to our usual experience, oblique orientations are most frequently encountered, then, after some period of time, responses should become repulsed away from oblique axes. To evaluate this hypothesis, we conducted two experiments that assessed whether repulsive response biases persisted when observers were exposed to a stimulus environment that was either consistent with the natural distribution of orientation or opposed to it (see Materials and Methods for details).

**Figure 5.**
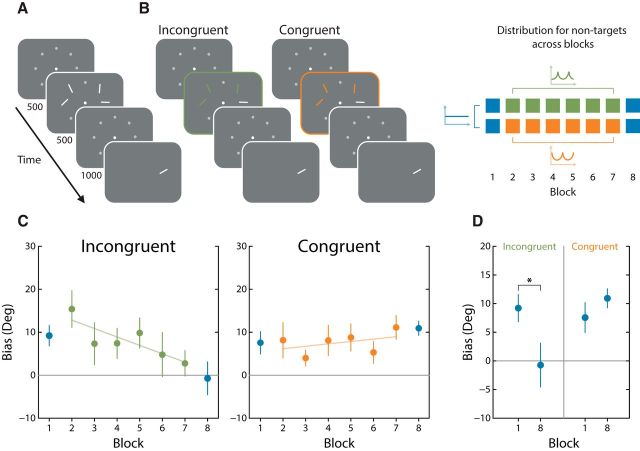
***A***, General experimental method. Observers reproduced orientations from arrays consisting of four oriented white lines. Across all blocks, targets were sampled from a uniform distribution. ***B***, The critical manipulation was the distribution of nontarget items during the adaptation blocks. In Experiment 1A, nontargets (green) were sampled from a bimodal distribution centered on oblique orientations (i.e., incongruent with the distribution in natural scenes), whereas in Experiment 1B, nontargets (orange) were sampled from a bimodal distribution centered on cardinal orientations (congruent with natural scenes). ***C***, Biases estimated from Experiment 1A (left) initially (block 1) indicated repulsion from the cardinals (positive bias). Bias steadily decreased across the adaptation blocks (blocks 2–7). During the postadaptation block (block 8), response biases were significantly less repulsed from cardinal orientations than preadaptation. Conversely, when the stimulus distribution was consistent with the distribution of orientation in nature (Experiment 1B, right), bias estimates remained unchanged by adaptation. ***D***, Comparisons between preadaptation and postadaptation biases highlight the significant reduction in repulsion away from the cardinal axes after exposure to the incongruent distribution only.

Specifically, observers completed an orientation reproduction task in which they were required to remember arrays of four white bars (Fig. 5*A*). Throughout six adaptation blocks, we manipulated the distribution of nontarget array items by sampling from one of two bimodal distributions: an incongruent distribution with peaks centered on oblique orientations and a congruent distribution with peaks centered on cardinal orientations. During preadaptation and postadaptation blocks, the nontarget distribution was uniform (Fig. 5*B*). We measured response biases for uniformly sampled target orientations presented over the eight blocks of trials. By manipulating only the distribution from which nontargets were sampled, we were able to measure response bias for feature values located along the entire orientation dimension while systematically altering the context in which target items were presented.

#### Experiment 1A: incongruent distribution

We first assessed whether changes in the frequency of nontarget orientations had an effect on bias estimates across adaptation blocks. Considering first the case where nontarget orientations were sampled from the incongruent distribution, there was an observable attenuation in the strength of repulsion away from the cardinal axes across adaptation blocks (slope, −0.034 ± 0.01; χ^2^_(1)_ = 6.63, *p* = 0.01; Fig. 5*C*, left). Critically, when the uniform sampling distribution was reintroduced after adaptation, the observed shrinkage in repulsion persisted, differing significantly from preadaptation levels of bias (*t*_(7)_ = 2.72, *p* = 0.029, *d* = 0.96; Fig. 5*D*). This change is consistent with the development of an increasing repulsive bias away from oblique orientations and suggests that subjects successfully integrated the changes in local orientation statistics.

#### Experiment 1B: congruent distribution

In comparison, when nontarget orientations were predominantly cardinal, response biases were virtually invariant across adaptation blocks (slope, 0.009 ± 0.01; χ^2^_(1)_ = 0.41, *p* = 0.52; Fig. 5*C*, right). There was no reliable difference between bias observed before and after adaptation (*t*_(7)_ = 1.01, *p* = 0.343, *d* = 0.36). As predicted by theory, consistency between local orientation statistics and the distribution assumed by the visual system resulted in very little change in response bias. Comparing across experiments, preadaptation and postadaptation biases exhibited a reliable distribution-by-block interaction effect (*F*_(1,14)_ = 7.32, *p* = 0.017), confirming that congruent and incongruent adaptations have different effects on bias.

## Discussion

We have presented an extended neural resource model that offers a principled explanation for the presence of stimulus-specific variation in orientation recall. Critically, achieving this result required only that the model know how orientation is distributed in nature, thus requiring no additional parameters to accommodate stimulus-specific effects. Fitting the model to data from three previous studies, we found it accurately reproduced observed changes in bias and precision with target orientation, and the modulating effects of set size on each measure, while still capturing observations from stimulus-averaged data such as the change of width and shape of error distributions with set size.

The model was then critically evaluated by testing how observers adapted to changes in the distribution of orientation. Despite the fact that biases in recall were seemingly in the wrong (anti-Bayesian) direction to have arisen from the influence of a prior, we found that they were altered by exposure to a new stimulus distribution in exactly the manner predicted by the efficient coding framework. Numerous studies have shown adaptation of Bayesian priors to changed stimulus environments occurring over short time scales (Adams et al., 2004; Körding and Wolpert, 2004; Berniker et al., 2010; Chalk et al., 2010). However, if adaptation were solely confined to the Bayesian decoding stage, then we would predict an increase in repulsion from the cardinals relative to preadaptation levels (in the incongruent condition). The fact that we instead observed a decrease in repulsion suggests that the tuning functions of the encoding population must also have adapted to the change in stimulus distribution. Rapid changes in tuning functions have been documented in visual neurons in response to adaptor stimuli presented in a cell's receptive field (Müller et al., 1999; Kohn and Movshon, 2004), but the kind of global adaptation of population tuning structure to reflect a new stimulus environment indicated by the present results has, to our knowledge, not been observed neurophysiologically. Nonetheless, our experimental results not only corroborate the proposed neural framework, but they lend further weight to the efficient coding hypothesis more generally.

The neural resource model was compared with an alternative that also claims to account for stimulus-specific effects: the modified slots-plus-averaging model (Pratte et al., 2017). Pratte et al. (2017) argued that, once supplemented with trigonometric functions that approximate stimulus-specific variations in bias and precision, the slots-plus-averaging model provided a better account of their data than one popular resource-based model, the variable precision model (van den Berg et al., 2012; but note the actual model tested differed in a number of respects from those authors' specification). Here we have shown that the neural resource model provides a consistently better description of data from three previous studies, including Pratte et al. (2017), than the modified slots-plus-averaging model. Perhaps more importantly, and unlike the neural resource model, the study by Pratte et al. (2017) did not provide any principled basis for the effects of stimulus-specific variation within the slot, or variable precision, framework. Though the neural resource model is broadly compatible with the variable precision model (in that both involve allocation of a continuous resource that determines mnemonic fidelity), the variable precision model is again primarily descriptive. It proposes that recall error distributions can be described by an infinite mixture of von Mises distributions, specified by a gamma distribution over Fisher information, and that the change in mean precision with set size can be approximated by a power law. Beyond this, the model does not provide any account of why the distributions, or the relationship with set size, take these particular forms.

In comparison, whereas the neural resource model provides only an algorithmic approximation to biological processes, the proposed mechanisms are far from arbitrary. Beyond the considerable evidence for population coding (Pouget et al., 2000, 2003), heterogeneity in orientation-selective neurons has also been observed in imaging and single-cell recording studies (Furmanski and Engel, 2000; Li et al., 2003). These found that a larger proportion of the population are devoted to representing cardinal orientations and that such neurons also possess narrower tuning curves than those encoding obliques. Given a parametric form for the probability density, it is possible to derive a heterogeneous population that mimics this cortical arrangement (Wei and Stocker, 2015), and we have shown that introducing this heterogeneity into the framework of population coding with normalization provides a very parsimonious account of stimulus-specific variation in working memory. Without a solid physiological foundation, it is difficult to imagine how either slot, or variable precision, frameworks could incorporate stimulus-specific variation in anything but an *ad hoc* manner.

One advantage of the efficient encoding framework is that stimulus-specific variation can be modeled across different feature dimensions and tasks (Wei and Stocker, 2017). For example, continuous report tasks that use color similarly treat hue as a circular random variable wherein the polar angle defines the gamut of the color space. Like orientation, it is often assumed that the distribution over polar angles is uniform, thereby implying a homogeneous population of hue-selective neurons. However, anisotropies in color recall precision have been documented and appear to be correlated across different color spaces (Bae et al., 2014), thus hinting at the possibility that hue is also represented efficiently. Determining the efficiency of color representations, though, is not straightforward because of the possibility that continuous hue representations are confounded with categorical information in recall data (Brouwer and Heeger, 2013; Hardman et al., 2017).

Processing of color stimuli is typically assumed to follow a bottom-up pathway (Gegenfurtner, 2003; Brouwer and Heeger, 2013), though top-down processes are also posited to have nontrivial effects on color perception (Witzel and Gegenfurtner, 2011; Hardman et al., 2017). Recent theoretical work has also shown how recurrent interactions between higher and lower cortical regions could qualitatively reproduce a number of category-based effects (Tajima et al., 2016). What remains unclear, however, is whether the abstraction of categorical information necessarily results from top-down processes or simply reflects some underlying regularity in the distribution of hue in nature. If the latter is true, then many of the color category effects that have been observed could be explained by a heterogeneous neural population that devotes a greater proportion of its resources to encoding category prototypes. In principle, such a scheme may be implemented within our modeling framework, though, ideally, this would be based on knowledge of how color signals are distributed in nature. Unfortunately, analysis of natural images has not provided a specific parametric distribution for hue (Cecchi et al., 2010; Kellner and Wachtler, 2013).

We acknowledge that our modeling framework is somewhat incomplete in that it does not allow for the possibility of swap errors. These errors arise when the feature value of a nontarget item is erroneously reported instead of the target value. The relative proportion of swap errors has been estimated using mixture models (Bays et al., 2009; van den Berg et al., 2014) and nonparametric methods (Bays, 2016), but these do not explain how the errors arise. Discrete capacity and variable precision models have not provided a principled account of swap errors. In contrast, recent work has shown that the neural resource model can quantitatively account for the empirical pattern of swap errors based on noise in neurons that encode conjunctions of stimulus features (Schneegans and Bays, 2017). Because the neurons in this model had homogeneous tuning functions, an important question for future work is how efficient coding influences the representation of feature conjunctions and thereby the frequency of swap errors.

One theoretical caveat with the neural resource model presented here is that it describes only the encoding and decoding of information and not its maintenance in the neural system. In reality, the maintenance of visual information is thought to rely on a balance of excitatory and inhibitory processes to sustain neural activity over time. One idea is that the maintenance of multiple items in memory is highly competitive owing to the limited supply of neural resources. Accordingly, information may be lost via activation bumps dissipating over time or merging with a nearby activation bump (Wei et al., 2012).

An alternative theory is that recurrent neural activity induces drift in the stored feature value, where the rate of drift leads to varying degrees of information loss over time (Burak and Fiete, 2012; Schneegans and Bays, 2018). This necessarily implies that greater amounts of drift would produce larger errors in feature report. An interesting question, then, is how recurrent activation might be affected by efficient coding and its associated effects on information loss. If the rate of drift is determined by the precision with which a representation can be decoded, as proposed by Burak and Fiete (2012), then one might intuit that because cardinal orientations are decoded more accurately, they will tend to drift less. This would be consistent with previous theoretical work indicating that structured heterogeneity can stabilize attractor networks (Kilpatrick et al., 2013). However, further work is required to relate these models to human memory performance.
